# Post-intensive care syndrome after a critical COVID-19: cohort study from a Belgian follow-up clinic

**DOI:** 10.1186/s13613-021-00910-9

**Published:** 2021-07-29

**Authors:** Anne-Françoise Rousseau, Pauline Minguet, Camille Colson, Isabelle Kellens, Sourour Chaabane, Pierre Delanaye, Etienne Cavalier, J. Geoffrey Chase, Bernard Lambermont, Benoit Misset

**Affiliations:** 1 Department of Intensive Care and Burn Center, University Hospital of Liège, Sart-Tilman B35, 4000 Liège, Belgium; 2 Department of Nephrology, University Hospital of Liège, Liège, Belgium; 3 Department of Clinical Chemistry, University Hospital of Liège, Liège, Belgium; 4 Department of Nephrology-Dialysis-Apheresis, Hôpital Universitaire Carémeau, Nimes, France; 5 Department of Mechanical Engineering, Centre for Bio-Engineering, University of Canterbury, Christchurch, New Zealand

**Keywords:** COVID-19, Critical care, Critical illness, Post-intensive care syndrome, Survivors, Survivorship, Outcome assessment, Health-related quality of life, Activities of daily living, Cognitive impairment, Post-traumatic stress disorder, Sleep

## Abstract

**Purpose:**

Many patients with coronavirus disease 2019 (COVID-19) required critical care. Mid-term outcomes of the survivors need to be assessed. The objective of this single-center cohort study was to describe their physical, cognitive, psychological, and biological outcomes at 3 months following intensive care unit (ICU)-discharge (M3).

**Patients and methods:**

All COVID-19 adults who survived an ICU stay ≥ 7 days and attended the M3 consultation at our multidisciplinary follow-up clinic were involved. They benefited from a standardized assessment, addressing health-related quality of life (EQ-5D-3L), sleep disorders (PSQI), and the three principal components of post-intensive care syndrome (PICS): physical status (Barthel index, handgrip and quadriceps strength), mental health disorders (HADS and IES-R), and cognitive impairment (MoCA). Biological parameters referred to C-reactive protein and creatinine.

**Results:**

Among the 92 patients admitted to our ICU for COVID-19, 42 survived a prolonged ICU stay and 32 (80%) attended the M3 follow-up visit. Their median age was 62 [49–68] years, 72% were male, and nearly half received inpatient rehabilitation following ICU discharge. At M3, 87.5% (28/32) had not regained their baseline level of daily activities. Only 6.2% (2/32) fully recovered, and had normal scores for the three MoCA, IES-R and Barthel scores. The main observed disorders were PSQI > 5 (75%, 24/32), MoCA < 26 (44%, 14/32), Barthel < 100 (31%, 10/32) and IES-R ≥ 33 (28%, 9/32). Combined disorders were observed in 13/32 (40.6%) of the patients. The EQ-5D-3L visual scale was rated at 71 [61–80]. A quarter of patients (8/32) demonstrated a persistent inflammation based on CRP blood level (9.3 [6.8–17.7] mg/L).

**Conclusion:**

The burden of severe COVID-19 and prolonged ICU stay was considerable in the present cohort after 3 months, affecting both functional status and biological parameters. These data are an argument on the need for closed follow-up for critically ill COVID-19 survivors.

**Supplementary Information:**

The online version contains supplementary material available at 10.1186/s13613-021-00910-9.

## Introduction

The health condition of survivors of a critical illness has become an increasing concern. Regardless of the primary disease, survivors of a prolonged stay in intensive care unit (ICU) may experience mid- and long-term morbidities related to the critical illness, the required support, and the environment. According to the princeps definition [1], the post-intensive care syndrome (PICS) refers to new or worsening physical (neuromuscular weakness and reduced autonomy for activities of daily living), mental (anxiety, depression, post-traumatic stress disorder [PTSD]) and neurocognitive disorders that negatively affect daily functioning and quality of life in survivors of critical illness. An expanded definition has recently been suggested, including additional factors, such as osteopenia, metabolic disorders, endocrine dysfunction, vulnerability, sleep disorders, chronic pain and fatigue [2]. Notably, ICU survivors have a higher risk of death in the years following discharge and a poorer quality of life compared to matched controls [3].

The coronavirus disease 2019 (COVID-19) can induce acute respiratory distress syndrome (ARDS), leading to ICU admission and prolonged ICU stay. It has been demonstrated COVID-19 ARDS follows the physiology of ARDS from other etiologies after establishing invasive ventilation [4]. Further recent improvement in COVID-19 ICU patient care has seen the number of survivors increase significantly [5], opening questions around mid- and long-term outcomes. Critical COVID-19 survivors may experience a range of sequelae, related to their critical condition (i.e. PICS) or the SARS-CoV-2 infection (i.e. the newly described post-acute COVID-19 syndrome, or PACS) [6]. Making allowances may be difficult, as there may be an overlap between symptoms.

A recent cohort study investigated PICS in a small number of critically ill COVID-19 survivors 1-month post-hospital discharge. Clinical assessment focused on limited outcomes (physical function, fatigue, insomnia, frailty, cognition, depression and PTSD) during a telehealth follow-up. More than 90% of patients reported symptoms affecting at least one major PICS domain [7]. Around 1 month after discharge, more than one third of the patients reported acute stress disorders [8] or cognitive dysfunction [9]. At 3 months following acute illness, survivors still reported an impaired health-related quality of life (HRQoL) [10, 11]. These results are also similar to previous ARDS cohorts [12]. At 6 months following a critical COVID-19, pain, discomfort, anxiety or depression were still prevalent, unlike mobility problems [13].

The aim of this study was to describe the mid-term outcomes and to assess the main PICS symptoms prevalence in critically ill COVID-19 survivors referred to a face-to-face consultation in our post-ICU follow-up clinic at 3 months following a prolonged ICU stay.

## Methods

### Participants: data sources

Since 2019, patients surviving an ICU stay ≥ 7 days are routinely invited to our post-intensive care follow-up clinic, at 1, 3, and 12 months following ICU discharge. A multidisciplinary team, including critical care physicians, critical care nurses, physiotherapists, dieticians, and psychologists, is involved at each time point. This face-to-face follow-up is standardized, addressing physical status and functional performances, nutritional status and body composition, bone health, mental health disorders, cognitive impairment, sleep disorders, and HRQoL. A blood analysis is also performed, focusing on inflammation and metabolic biomarkers. Measurement of C-reactive protein (CRP) and creatinine is part of our standard analysis.

All consecutive critically ill COVID-19 patients admitted to ICU during the first wave (from March 1st to July 17th, 2020, this late date coinciding with a radical change in COVID-19 treatment including steroids and a broader use of high flow nasal oxygen) were invited to attend the consultations. Patients were not included if they were still hospitalized in an inpatient rehabilitation facility, or if they were unable to communicate in the French language, the local language. Clinical data about physical, mental and neurocognitive status, as well as biological parameters related to inflammation status and kidney function, were prospectively collected following attendance at the clinic, 3 months (M3) after ICU discharge. Demographic data and data related to the ICU stay were collected retrospectively and extracted from the medical charts.

In accordance with Belgian law, informed consent was not required because the study did not modify patients’ management and the data were anonymously collected. This interpretation was confirmed by the Ethics Committee of the University Hospital of Liege (local reference 2020/424).

### Description of the study intensive care department

Our 6-unit adult intensive care department is located in a university hospital, and includes 44 beds. It also includes 6 beds in a dedicated burn intensive care unit. The medical team includes 12 senior trained physicians and 22 residents. Each unit is staffed with a physiotherapist. The number of patients per nurse varies from 2/1 in the morning shift to 3/1 during the afternoon and the night shifts. Previously to the pandemic, from mid-October 2019 to the end of February 2020, 977 patients and 28 burn patients were admitted to our ICUs. At the peak of the first wave, during March and April 2020, access for burn patients at our burn unit was temporarily suspended, and our 50 ICU beds were thus entirely dedicated to COVID-19 patients. Ten additional beds in the recovery room were dedicated to general critical care. The 60 beds had a mechanical ventilation capacity. Additional physicians and nurses were assigned in each unit or during each shift respectively, according to the hospital means.

### Clinical variables (Additional file 1)

The Montreal Cognitive Assessment (MoCA) was used to examine global cognitive function, including visuospatial, executive function, attention/working memory, episodic memory, and language. The MoCA total score was used for analysis: it ranges from 0 to 30, the lower scores indicating worse cognitive performances. The validated cut-off of 26 was used to distinguished light cognitive disorders (≥ 26) and proven cognitive impairments (< 26) [14].

Mental health status was assessed using the Hospital Anxiety and Depression scale (HADS) and the impact of event scale-revised (IES-R). The HADS consists of two 7-item subscales evaluating symptoms of depression (seven items—HADS-D subscale) and symptoms of anxiety (seven items—HADS-A subscale) [15]. The standard cutoff threshold value of > 7 out of 21 on either subscale was used to define a borderline status (score 8 to 10) or clinically significant status (score 11 to 21) of depression or anxiety, respectively. The IES-R is a 22-item tool that detects symptoms indicating a post-traumatic stress disorder (PTSD) [16]. It measures the severity of the three categories of PTSD symptoms: avoidance, intrusion and hyperarousal symptoms. A cutoff score ≥ 33 out of 88 was adopted to indicate severe psychological impact of the traumatic event.

The Pittsburgh Sleep Quality Index (PSQI) is a validated tool used to obtain self-reported sleep quality [17]. The PSQI contains 19 self-rated questions, evaluating subjective sleep quality, sleep latency, sleep duration, habitual sleep efficiency, sleep disturbances, use of sleep medications and daytime dysfunction over the previous month. Each component generates a subscale score of 0–3, with 0 indicating no difficulty, and 3 indicating severe difficulty. These 7 scores are combined in one global score of 0–21 points (0 = no difficulty, 21 = severe difficulty) where a score of ≥ 5 indicates poor sleep quality.

The Barthel Index of activities of daily living (ADL) was used to measure functional status and dependency. It consists of 10 subheadings as feeding, bathing, grooming, dressing, bladder control, bowel control, toilet use, chair–bed transfer, mobility and stair climbing [18]. Scoring ranges from 0 to 100: a score of 100 is defined as being capable of ADL complete self-care.

Peripheral muscle strength was determined using handgrip and quadriceps dynamometry. Handgrip strength was assessed using a Jamar hydraulic hand dynamometry. Measurements were performed in a sitting position, with the elbow in 90° flexion. The protocol consisted of three consecutive maximal contractions for each muscle group, preceded by three warm-up trials. Observers provided standardized encouragement. The three measurements were performed with 30-s intervals between contractions. Subjects were asked to gradually increase their muscle force to a maximum effort which had to be sustained for 6 s. The highest performance was considered for analysis. Grip strength varies with age and sex: strength ≥ 25 kg in men ≤ 60 years and ≥ 23 kg in men between 61 and 79 years or strength ≥ 14 kg in women ≤ 60 years and ≥ 13 kg in women between 61 and 79 years are considered normal [19]. Maximal isometric quadriceps strength was measured with a MicroFET2 hand-held dynamometer (Hoggan Indiustries, Inc., West Jordan, UT, USA), with the patient lying in supine position. The highly standardized testing protocol is detailed in a previously published validation study [20]. Magnitude of the values that can be observed in healthy and critically ill patients are described elsewhere [21]. To reduce inter-individual variability and minimize the effect of subject weight on muscle strength, absolute strength was normalized according to actual body weight (expressed in N/kg). Unfortunately, at present, no cut-off values for quadriceps weakness are defined in critical care populations using the above-described protocol.

HRQoL was measured using the EQ-5D-3L. This tool comprises two sections: a five-question descriptive component which explores five dimensions: mobility, self-care, usual activities, pain/discomfort and anxiety/depression. Each question has three possible answers, rated from 1 to 3: no problems, some problems and extreme problems. The second section is a visual analogue scale (EQ VAS) about HRQoL.

Finally, patients were questioned about their living condition, return to previous level of activities (employment or leisure activities in unemployed patients), ongoing outpatient physiotherapy at M3, and emergency department visits between hospital discharge and M3.

### Biological variables

The biological data were generated from one single laboratory (Unilab, CHU de Liège) accredited for ISO 15,189 Guideline. The following variables were collected: serum CRP and serum creatinine. The normal range is 0–5 mg/L for CRP, 0.55–1.02 mg/dL for creatinine in females and 0.55–1.18 mg/dL in males. The glomerular filtration rate (eGFR) was estimated using MDRD equation during ICU stay, and using both creatinine-based CKD-EPI equations at M3.

### Analysis

We arbitrarily used the Barthel Index, MoCA and IES-R to sum up the three components of the PICS (according to the princeps definition) and to analyze its prevalence.

Patients were separated into two groups at M3: patients who were discharged directly from hospital to their home, and patients who were discharged from hospital to inpatient rehabilitation facilities. The above-described outcomes were compared between groups.

### Statistical analysis

Statistical analysis was performed using Graphpad Prism (version 9.0 for Mac OSX, Graphpad Inc., San Diego, CA, USA)*.* Quantitative variables were expressed using median and interquartile range, and qualitative variables were described using count and percent. Non-parametric tests were chosen due to the small sample size. Comparisons between groups were made using Chi‐square test for categorical variables and using Mann–Whitney for continuous variables. Correlation between parameters was assessed using nonparametric Spearman test. A *p* value < 0.05 was considered statistically significant.

## Results

**Fig. 1:**
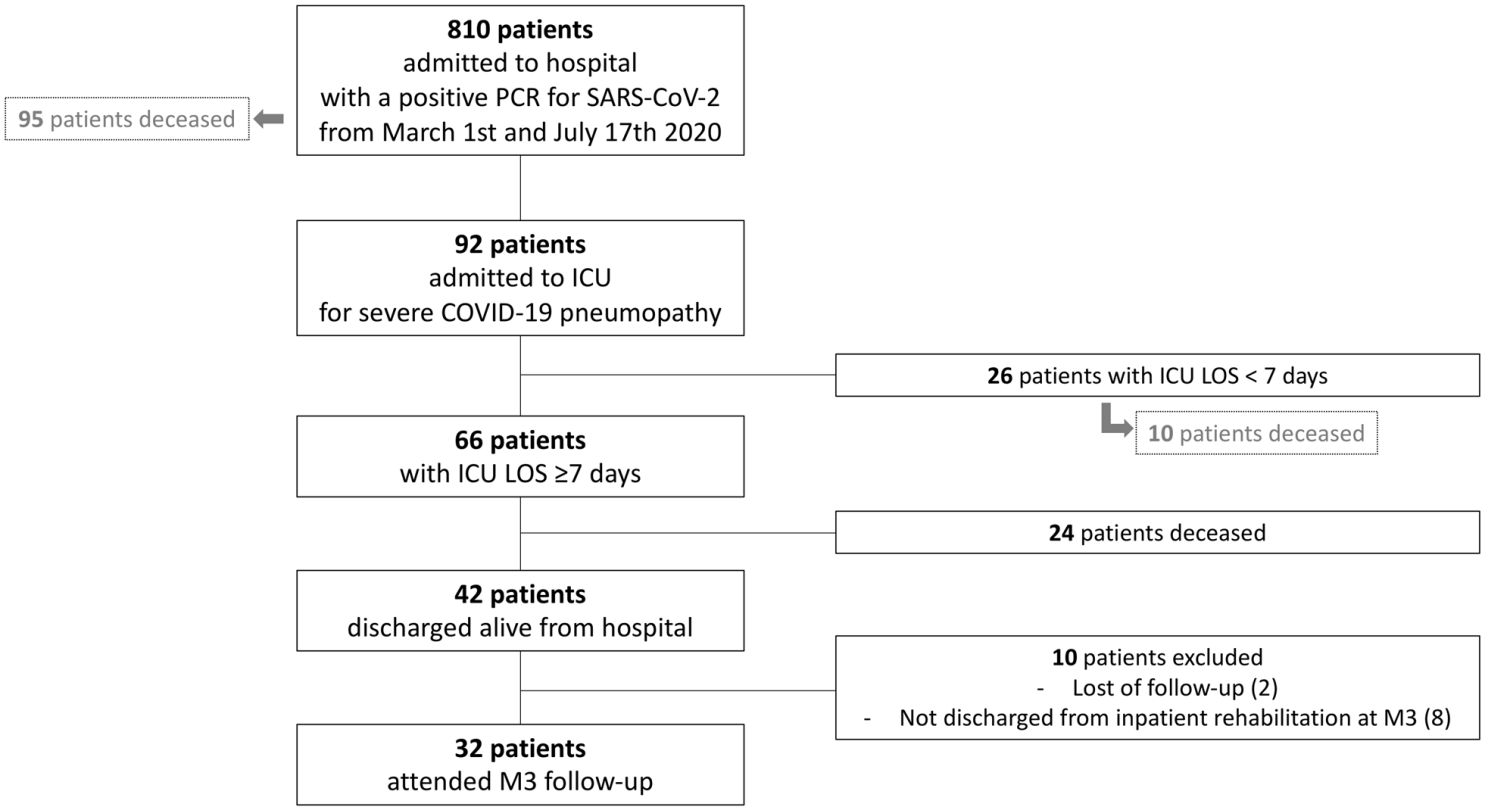
Flowchart. *ICU *intensive care unit, *LOS* length of stay, *PCR* polymerase chain reaction

From March 1st, 2020, until July 17th, 2020, 810 patients were admitted to our hospital with a positive polymerase chain reaction for SARS-CoV-2, and 92 patients were admitted to ICU for severe COVID-19 pneumonia. Of the 66 patients who spent 7 days or more in ICU, 42 patients were discharged alive from the hospital. Eight of these patients were still hospitalized for rehabilitation at M3, and 2 patients were lost to follow-up. Finally, 32 patients attended the M3 follow-up visit at our post-intensive care follow-up clinic and were analyzed (Fig. 1). Descriptive characteristics of the included subjects are detailed in Table 1. Twenty-one patients (65.6%) were aged < 65 years and 43.8% were retired (14/32 patients). The majority of the patients were mechanically ventilated for a long period and had a prolonged ICU and hospital length of stay (LOS).

**Table 1:** M3 cohort demographics

Data	COVID-19 ICU cohort *n* = 92	ICU patients deceased at hospital *n* = 31	M3 cohort *n* = 32
Age, years	65 [54–71]	70 [60–79]	62 [49–68]
Male, *n* (%)	63 (68.5)	23 (74.2)	23 (72)
Weight, kg	93 [80–101]	93 [80–105]	99 [86–105]
Height, cm	173 [165–180]	172 [167–180]	173 [164–180]
BMI, kg/m^2^	30.9 [27–34]	31.1 [26.1–35]	31.5 [30–34.8]
Comorbidities			
Diabetes	37 (40.2)	15 (48.4)	20 (62.5)
Hypertension	54 (58.7)	21 (67.7)	17 (53.1)
Cardiac^a^	35 (38)	16 (51.6)	10 (31.2)
Respiratory^b^	16 (17.4)	8 (25.8)	4 (12.5)
Chronic kidney disease	18 (19.6)	12 (38.7)	2 (6.2)
Immunosuppression	5 (5.4)	3 (9.7)	1 (3.1)
SOFA at admission	6 [3–8]	7 [5–8]	7 [4–8]
SAPS II	36 [28–47]	42 [33–54]	37.5 [30.5–44]
Mechanical ventilation, *n* (%)	71 (77.2)	28 (90.3)	30 (93.7)
Duration of mechanical ventilation, days	17 [11–26]	13 [6–23]	21 [12–29]
PaO_2_/FiO_2_ at admission	–	–	97.5 [74.1–131]
Worse PaO_2_/FiO_2_	–	–	71.1 [62.1–81.1]
Vasopressive support, *n* (%)	56 (60.9)	24 (77.4)	27 (84.4)
Duration of norepinephrine administration, days	17 [11–26]	4 [1–13]	5 [2–10.5]
Peak creatinine > upper reference value, *n* (%)	–	–	23 (71.8)
Peak creatinine, mg/dL	–	–	1.7 [1–4.1]
Worse eGFR (MDRD), ml/min/1.73 m^2^	–	–	40.7 [14.4–65.8]
Renal replacement therapy, n (%)	18 (19.6)	10 (32.3)	5 (15.6)
Extracorporeal membrane oxygenation, n (%)	3 (3.3)	3 (9.7)	0
Peak C-reactive protein, mg/L	–	–	306.4 [253.1–363.2]
ICU LOS, days	16 [7–28]	13 [7–23]	23 [15–39]
Hospital LOS, days	19 [12–28]	14.4 [9–24]	40 [29–52]
Hospital mortality, *n* (%)	31 (33.7)	31 (100)	–
Destination at hospital discharge			
Home, *n* (%)	–	–	15 (46.9)
Rehabilitation facility, *n* (%)	–	–	17 (53.1)
Rehabilitation center LOS, days	–	–	30 [20–40]

*BMI* body mass index, *ICU* intensive care unit, *LOS* length of stay, *SAPS II* simplified acute physiology score, *SOFA* sequential organ failure assessment

^a^Ischemic heart disease, valvular disease, cardiomyopathies, and chronic heart disease

^b^Asthma, chronic obstructive pulmonary disease and interstitial lung diseases

At M3, 28/32 patients (87.5%) did not return to their previous level of activity, either employment for previously active patients or leisure for unemployed or retired patients. From hospital discharge until the M3 visit, 4/32 (12.5%) patients visited an emergency department, at least one time.

**Fig. 2:**
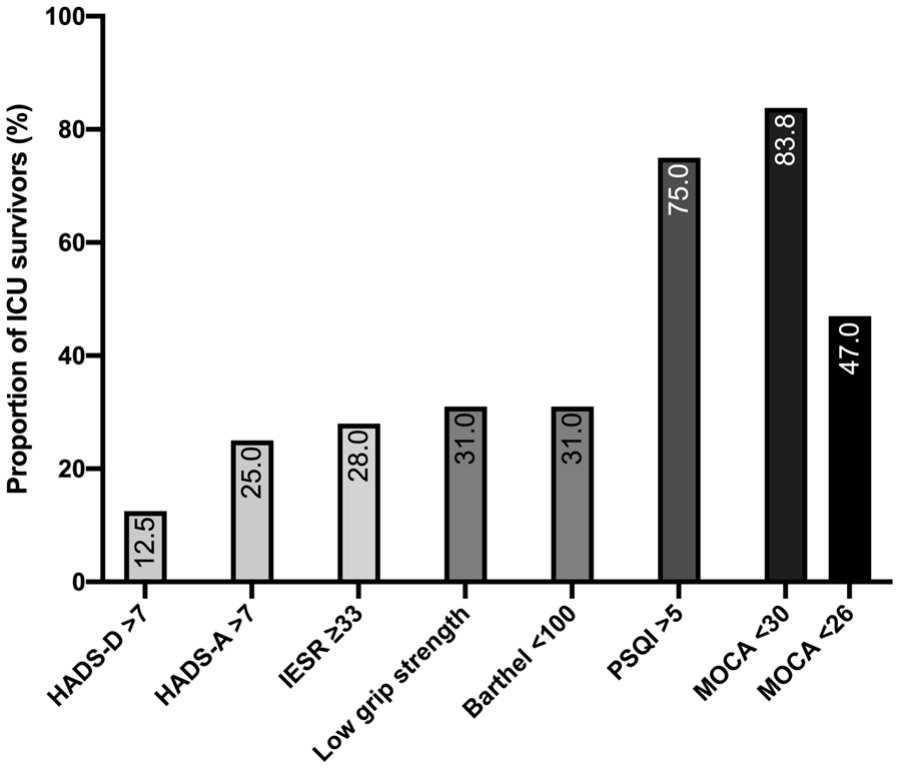
Proportion of survivors with abnormal results to the questionnaires

**Fig. 3:**
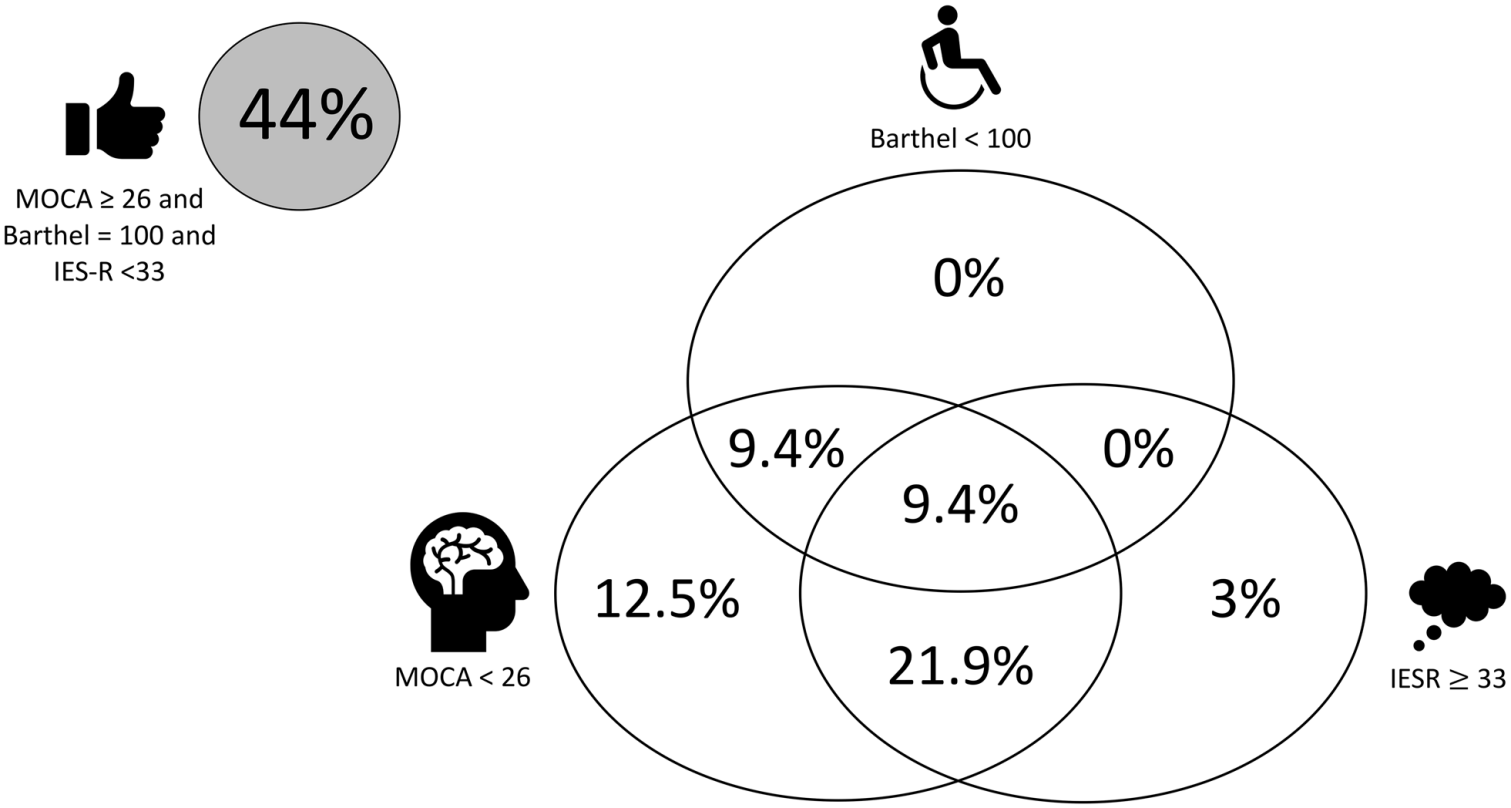
Proportion of patients experiencing none (grey circle), one or a combination (white circles) of the three components of the post-intensive care syndrome (PICS), analyzed using Barthel Index, MoCA and IES-R (as defined in “Methods”)

The M3 visit occurred 94 [90–101] days after ICU discharge. The M3 assessment is detailed in Table 2. The proportion of patients with abnormal results to the questionnaires and tests are shown in Fig. 2. Sleep quality was poor in a vast majority of the ICU survivors, mainly marked by sleep fragmentation and frequent arousals from sleep. The proportion of patients experiencing one or a combination of the key features of the PICS is detailed in Fig. 3. In the studied population, 43.8% (14/32) had MoCA, IES-R and Barthel in acceptable ranges. Only 2/32 (6.2%) patients had fully normal scores for the three MoCA, IES-R and Barthel scores, and were considered as having fully recovered. A quarter of patients (8/32) demonstrated a persistent inflammation based on CRP blood level (CRP 9.3 [6.8–17.7] mg/L). At M3, 9/32 (28%) had an estimated GFR < 60 ml/min/1.73 m^2^ based on CKD-EPI creatinine equation (47) [35.5–54.5] ml/min/1.73 m^2^.

Table 3 depicts the comparison of demographics and outcomes in patients who went to a rehabilitation center after hospital discharge, in relation to those who did not. These latter patients, while having similar severity score on ICU admission (*p* = 0.596), had significant shorter duration of mechanical ventilation (*p* = 0.011) and ICU LOS (*p* = 0.004). Patients who benefited from rehabilitation recovered similar cognitive function, mental health, quadriceps strength and HRQoL at M3, but were still significantly more dependent (*p* = 0.03), and had a significantly lower handgrip strength at M3 (*p* = 0.005), when compared to patients who were discharged from hospital at home. In the two groups, a similar proportion of patients had an ongoing physiotherapy in an outpatient setting at M3.

**Table 2:** M3 assessment

Data	*n* = 32
MoCA	27 [24–28]
HADS-A	4 [1–6]
HADS-D	1 [0–3]
IES-R	11 [4–24]
PSQI	6 [4–11]
EQ-5D score	6 [6–8]
EQ-5D visual analogic scale	71 [61–80]
Barthel Index	100 [100–100]
Handgrip strength (kg)	28 [21–37]
Quadriceps strength (N)	261 [191–338]
Quadriceps strength (N/kg)	2.9 [2.3–3.5]
C-reactive protein (mg/L)	2.6 [1.7–6.2]
Serum creatinine (mg/dL)	0.96 [0.75–1.23]

**Table 3:** Demographics and outcomes in patients who benefited or not from an inpatient rehabilitation after hospital discharge

Data	Rehabilitation (*n* = 17)	No rehabilitation (*n* = 15)	*p* value
Age, years	62 [47–69]	63 [50–67]	NS
SOFA	7 [3–9]	6 [4–7]	NS
Duration of mechanical ventilation, days	24 [18–32]	16 [11–26]	0.004
ICU LOS, days	39 [23–43]	16 [11–26]	0.011
MoCA	26 [23–28]	27 [22–28]	NS
IES-R	6 [4–24]	9 [3–34]	NS
EQ-5D score	7 [6–9.5]	6 [5.7–7.2]	NS
EQ-5D visual analogic scale	70 [65–80]	75 [64–80]	NS
Barthel Index	100 [75–100]	100 [100–100]	0.03
Handgrip strength (kg)	21 [14–27]	32 [28–42]	0.005
Quadriceps strength (N)	241 [217–268]	332 [182–365]	NS
Quadriceps strength (N/kg)	2.7 [2.3–3.1]	3 [2.3–4.3]	NS

*BMI* body mass index, *ICU* intensive care unit, *LOS* length of stay, *SOFA* sequential organ failure assessment

## Discussion

The burden of a severe COVID-19 and a prolonged ICU stay was considerable in the present cohort, affecting both functional status and biological parameters in, respectively, a half and a quarter of patients. Less than 10% of patients presented no PICS symptoms and were fully recovered at M3. Similar observations were already reported at 1 month in recent studies [7–9], suggesting very slow improvement in clinical status without active and continuous post-ICU care. The actual consequences for survivors may be underestimated, given the inherent selection bias of a follow-up clinic, where only the fittest patients accept or are able to attend the follow-up consultations. In the present first wave cohort of COVID-19 critically ill patients, up to 20% were still hospitalized in an inpatient rehabilitation facility and thus were not assessed at M3.

The observations in the present critical COVID-19 cohort are similar to the few comparable results already published. Similar EQ-5D-3L scores were described at M3 in a small cohort of patients who experienced a slightly shorter duration of mechanical ventilation and ICU LOS [10]. In a large cohort of patients who spent about 10 days in ICU, sleep disorders and cognitive impairments were the two most frequent sequelae reported by survivors four months following discharge [11].

Classically, up to 80% patients surviving non-COVID-19 ARDS are anticipated to present at least one symptom of PICS [22]. This same proportion was confirmed in our present COVID-19 cohort. We also observed similar prevalence of mid-term disorders when compared to previously published data about non-COVID-19 critically ill survivors, at the same evaluation time point (M3). A recent systematic review suggested a high prevalence of cognitive disorders in ICU survivors: about 50 to 80% of the ICU survivors presented cognitive impairments. The prevalence was higher in ARDS survivors compared to mixed ICU patients, and was also higher when using objective assessment tools, compared to subjective assessment [23]. PTSD has been observed in 14 to 36% of medical ICU survivors [24, 25]. One year after discharge, a higher dependency was observed in patients between 65 and 74 years who survived a mean ICU LOS of 10 days had a higher dependency, compared to baseline: their Barthel Index score did not reach 90 out of 100 [26]. Sleep disorders and circadian disruption were equally common in survivors of non-COVID-19 acute respiratory failure as in the present cohort, impacting three quarters of the patients [27]. Finally, two thirds of non-COVID-19 ICU survivors experienced a decrease in employment, when previously employed prior to their acute illness [28]. These data suggest critically ill COVID-19 survivors are not so different from other ICU survivors, at least regarding these M3 mid-term outcomes.

Persistent systemic inflammation, based on CRP levels, has been described in at least 25% of ICU survivors at 3 months following discharged and was associated with impaired physical recovery [29]. This relationship was not formally analyzed in the present cohort due to its small size. However, a similar proportion of patients with persistent inflammation was observed.

Multidisciplinary rehabilitation of critically ill COVID-19 survivors is recommended by experts, with the objective to reduce the long-term complications [30, 31]. In the present cohort, patients who received inpatient rehabilitation had a longer duration of mechanical ventilation and a longer ICU LOS. Compared to patients who were not transferred to such facilities, they had similar HRQoL, cognitive and psychological outcomes at 3 months following ICU discharge. Despite similar quadriceps strength, their handgrip strength and autonomy were still lower. No strong conclusion can be drawn from these observations, as the unknown baseline status and the different ICU characteristics between the two groups could have impacted their M3 status, independently of the rehabilitation. The effects of enhanced physical rehabilitation following ICU discharge on functional outcomes and mortality are controversial [32], mainly due to a large variability in studied rehab program (type and timing), patients (age, baseline status) and outcomes (type and timing). The benefits of such rehabilitation should probably be considered at an individual level rather than at a group level.

This study extends the knowledge regarding mid-term outcomes in critically ill COVID-19 survivors, throughout a comprehensive assessment in a homogeneous population of the first wave of the pandemic. These data can help highlight this growing and potentially hidden public health issue, as the world wrestles with ongoing COVID-19 infections and other critical illnesses. However, some limitations need to be acknowledged. First, the sample was limited and the cohort was monocentric. However, 32 (80%) of eligible patients attended the follow-up, and the sample was representative of the cohort. Further studies or meta-analyses will be required to confirm and add further follow-up over time to these initial results, where there appears to be only a single prior result. Post-ICU follow-up clinics are still rare in Belgium, due to economic and organizational barriers. In other countries, they exist in various forms, with different programs of consultations, outcome assessments and rehabilitation strategies. Most often, they are part of experimental setups. In such context, building a multicentric study was difficult during the first wave of the pandemic and the associated hospital and social disruptions. As a result, we did not search for specific clinical risk factors to develop PICS symptoms due to the small cohort size and variability in how follow-up clinics are implemented, and such statistical analysis remains for a larger, future study. Second, this study lacks precise assessment of baseline clinical status. It is a common issue with many studies assessing long-term outcomes in ICU survivors in general, and is related to the unpredictable characteristic of ICU admissions, particularly during this pandemic. This pitfall can lead to misinterpretation of what is considered as post-intensive care sequelae. Third, there was no non-COVID-19 control group. When compared with published non-COVID survivors of ARDS, we found similar outcomes in the present cohort. However, it will be interesting to compare prevalence and severity of disorders in COVID-19 and non-COVID-19 ARDS survivors in further studies. Finally, the present results applied only to first wave patients, where significant changes in care were implemented between waves in our center, and similarly in others. Comparisons of similar outcomes and patients between the first and second-wave cohorts will undoubtedly provide further assessment of the impact of these clinical changes on outcomes. These analyses are currently ongoing in our center.

## Conclusion

The present study is one of the first studies focusing on expanded PICS and characterizing mid-term physical, functional, cognitive, mental and biological outcomes in critically ill COVID-19 survivors. Three months after ICU discharge, a huge proportion of patients experienced single or combined symptoms of the PICS, according to its princeps definition. The ICU burden mainly impacted cognitive function, sleep and autonomy for activities of daily living. The vast majority of the patients did not get their baseline level of activities back. These data are an argument on the need for closed follow-up for critically ill COVID-19 survivors.

## Supplementary Information

**Additional file 1:** **Table S1**. Measurement tools used at M3, with their normal ranges and abnormal thresholds if available.

## Data Availability

The datasets used and/or analyzed during the current study are available from the corresponding author on reasonable request.

## Abbreviations

ADL: Activities of daily living
AKI: Acute kidney injury
ARDS: Acute respiratory distress syndrome
CKD: Chronic kidney disease
CRP: C-reactive protein
GFR: Glomerular filtration rate
HADS: Hospital anxiety and depression scale
HRQoL: Health-related quality of life
ICU: Intensive care unit
ICU-AW: Intensive care unit-acquired weakness
IES-R: Impact of event scale-revised
LOS: Length of stay
MoCA: Montreal cognitive assessment
PICS: Post-intensive care syndrome
PSQI: Pittsburgh Sleep Quality Index
PTSD: Post-traumatic stress disorder
