# Supplementary material for: Post-intensive care syndrome after a critical COVID-19: cohort study from a Belgian follow-up clinic
Source: Ann Intensive Care. 2021 Jul 29;11:118. doi: 10.1186/s13613-021-00910-9 (PMC8319705; doi:10.1186/s13613-021-00910-9)
Supplement: Supplementary file 1 — Additional file 1: Table S1. Measurement tools used at M3, with their normal ranges and abnormal thresholds if available. [file 13613_2021_910_MOESM1_ESM.docx]

**Additional file 1**

**Table S1: Measurement tools used at M3, with their normal ranges and abnormal thresholds if available.**

*NA: not available*

| Tests and scores | Ranges | Abnormal thresholds |
| --- | --- | --- |
| MoCA | 0 – 30 | 18-25 = mild cognitive impairment  10-17 = moderate cognitive impairment  < 10 = severe cognitive impairment |
| HADS-A | 0 - 21 | > 7: borderline status  ≥ 11: anxiety |
| HADS-D | 0 - 21 | > 7: borderline status  ≥ 11: depression |
| IES-R | 0 - 88 | ≥ 33: PTSD |
| PSQI | 0 - 21 | ≥ 5: poor sleep quality |
| EQ-5D-3L score | 5 - 15 | NA |
| EQ-5D visual analogic scale | 0 - 100 | NA |
| Barthel index | 0 - 100 | 91-99 = slight dependency  61-90 = moderate dependency  21-60 = severe dependency  0-20 = total dependency |
| Handgrip strength | ≥ 25kg in men ≤ 60 years  ≥ 23kg in men between 61 and 79 years  ≥ 14kg in women ≤ 60 years  ≥ 13 kg in women between 61 and 79 years | 11 kg in males, > 7 kg in females |
| Quadriceps strength | NA | NA |
| C-reactive protein | 0 – 5 mg/L | > 5mg/L |
| Serum creatinine | 0.55-1.18mg/dL in males  0.55-1.02 mg/dL in females | > 1.18mg/dL in males  > 1.02 mg/dL in females |
| Serum cystatin C | 0.62 – 1.11 mg/L | > 1.11 mg/L |
